# The mediating role of organizational justice in the relationship between value-centered school culture and organizational citizenship: A case of North Cyprus

**DOI:** 10.1371/journal.pone.0354841

**Published:** 2026-07-28

**Authors:** Cemaliye Karadayı Numan, Yalın Kılıç, Hülya Şenol

**Affiliations:** 1 Cyprus Aydın University, Ozanköy, Kyrenia, North Cyprus; 2 Cyprus Health and Social Sciences University, Education Administration and Inspection Department, Kutlu Adalı Bulvarı, Güzelyurt, Turkey; Sultan Qaboos University and Ain Shams University, OMAN

## Abstract

This quantitative research aimed to determine the mediating role of organizational justice in the relationship between teachers’ perceptions of value-centered school culture and their views on organizational citizenship. A relational screening model was utilized as the research framework. A survey was administered to 301 randomly selected teachers from general secondary education in the two largest cities of North Cyprus, Nicosia and Kyrenia. The survey consisted of four parts: A personal information form,Value-Centered School Culture Scale, Organizational Justice Scale, and Organizational Citizenship Scale. As the data showed a normal distribution, parametric tests were performed to analyze the data. In addition, the mediating role of the Organizational Justice Scale in the relationship between the participating teachers’ Values-Centered School Culture Scale scores and the Organizational Citizenship Scale scores was examined using observed-variable path analysis. The findings of this research indicated that teachers’ perceptions of values-centered school culture,organizational citizenship, and organizational justice were above average. Significant positive correlations were identified between organizational justice and organizational citizenship, as well as between organizational citizenship and a values-centered school culture. Observed-variable path analysis revealed that a values-centered school culture significantly and positively predicted organizational justice, which in turn significantly and positively predicted organizational citizenship. In addition, values-centered school culture is associated with organizational citizenship. This study offers empirical evidence that a values-centered school culture is a crucial organizational factor associated with teachers’ perceptions of organizational justice and organizational citizenship. The findings reveal that values-centered school culture has both direct and indirect effects on organizational citizenship, mediated by organizational justice. This underscores the significant role of school culture in relation to teachers’ perceptions and behaviors regarding their organization. These results expand existing knowledge by illustrating the interconnectedness of organizational culture, justice, and citizenship within educational settings. Additionally, organizational-level characteristics should be taken into account when examining teachers’ work-related attitudes and behaviors.

## Introduction

The culture of an organization consists of its dominant and shared values, beliefs, stories, and slogans. These elements create symbolic meanings for employees [1] and help distinguish an organization from others. The culture acts as a control mechanism that shapes the attitudes and behaviors of employees [2]. Values are a set of standards intrinsic to individuals that differentiate them from others by shaping and guiding their goals, choices, actions, ideals, and thought patterns [3]. A value-centered school culture refers to the adoption and implementation of these values by all school staff and administrators, fostering a balanced environment. This approach also highlights the importance of involving families within the school context [4]. School culture is associated with teachers’ organizational citizenship behaviors. The levels of organizational citizenship among teachers are related to outcomes such as efficiency and student engagement [5]. Organizational Citizenship Behavior of employees enhances organizational performance and increases productivity [6,7], lowers staff intention to leave [8]. Organizational justice is closely related to value systems and culture in organizations. The main studies examining these relationships in the literature after the 2000s are: organizational culture [9], value systems [10], values [11], management according to values [12].

There are studies that showed the relationship between organizational citizenship and culture [13–19]; organizational culture and justice [20–26]; organizational justice and organizational citizenship [27–38].

Although there are many studies examining the relationships between organizational culture, organizational citizenship and organizational justice in the business management literature, the rare studies examining teachers’ perceptions of school culture, organizational justice and citizenship together are the studies conducted by [39] and [40]. [39] found the moderating role of organizational culture in the relationship between justice perceptions and organizational citizenship behavior as perceived by the lecturers at universities. The results of this study indicated that there is a strong relationship between organizational citizenship behavior and interactional justice in organizations that emphasize respect for individuals. Conversely, organizations that have a higher focus on team orientation exhibit a weaker relationship between procedural justice, distributive and organizational citizenship behavior. [40] found a moderate positive relationship between organizational justice and organizational culture among the variables that positively affect teachers’ organizational citizenship behaviors. In addition, there is no study in the national and international literature examining the relationships between value-centered school culture, organizational citizenship and organizational justice according to teachers’ perceptions. This study intends to fill this gap in the literature and will offer an empirical evidence that a values-centered school culture is a crucial organizational factor influencing teachers’ perceptions of organizational justice and organizational citizenship and expand existing knowledge by illustrating the interconnectedness of organizational culture, justice, and citizenship within educational settings. In this light, this research aimed to examine the mediating role of organizational justice in relationship between value centered school culture and organizational citizenship.

### Theoretical framework

Social Exchange Theory (SET), introduced by [41], offers a strong theoretical framework for understanding discretionary employee behaviors that result from reciprocal relationships within organizations. According to SET, individuals engage in social interactions with the expectation that favorable treatment will be returned over time. In an organizational context, this means that when employees feel they are treated fairly and equitably by their organization, they tend to respond with positive attitudes and behaviors that go beyond their formal job requirements. Organizational justice refers to employees’ perceptions of fairness regarding workplace procedures, interactions, and outcomes. Based on Social Exchange Theory (SET), these perceptions of justice are crucial as they signal respect, trust, and support from the employer. When employees feel that they are treated fairly, they are more likely to reciprocate through organizational citizenship behaviors (OCB). These behaviors include helping colleagues, being conscientious, and showing loyalty to the organization [42,43]. Such discretionary actions are a form of social repayment within the exchange relationship. Fair treatment enhances employees’ intrinsic motivation and commitment, thereby strengthening their willingness to engage in organizational citizenship behaviors [44,45]. Thus, Social Exchange Theory offers a clear explanatory mechanism linking organizational justice to organizational citizenship behavior by emphasizing reciprocity as the underlying driver of employee behavior. Based on this theoretical reasoning, the present study proposes that organizational justice positively and significantly predicts organizational citizenship behavior.

### Value-centered school culture

School culture encompasses the shared values, beliefs, norms, traditions, and practices that affect how members of the school community think, feel, behave, and interactions among teachers, students, administrators, and other stakeholders, and plays a crucial role in shaping teaching practices, student learning, and the overall effectiveness of the school [46,47]. School culture is associated with the actions, thoughts, and feelings of educational professionals [48], flowing from administrators and teachers to students [49], organizational structures, the broader social and community context in which the school operates, shared values and beliefs, leadership practices,and teacher collaboration [50]. Values education is the deliberate process by which schools promote the moral, ethical, social, and civic values that guide students’ behaviour, decision-making, and interactions with others. The aim is to foster qualities such as respect, responsibility, honesty, empathy, and fairness, enabling learners to develop character alongside academic competence. Values education is embedded in the formal curriculum, school culture, teacher behaviour, and everyday interactions, making it a holistic and continuous process [51]. By nurturing shared values, schools contribute to students’ personal development, social cohesion, and responsible citizenship all of which are essential for sustaining democratic and inclusive societies [52].

The Living Values Education Program was originated from an international project developed by the Brahma Kumaris in 1995 to celebrate the 50th anniversary of the United Nations. This initiative, titled “Sharing Our Values for a Better World,” focused on 12 universal values: happiness, honesty, humility, cooperation, freedom, love, peace, respect, responsibility, simplicity, tolerance, and unity. The project gained significant attention in Turkey, as well as in many other countries, and was later implemented as a “Values and Education Project” after being adopted by the decisions of the 18th National Education Council. The Living Values Education Program was launched in 2012 as a framework for values education in schools and has since been implemented in various regions of Turkey [53]. Values education varies from country to country. For example, in Australia, the national values education framework includes nine core values: sensitivity and compassion, striving to do one’s best, fairness, freedom, honesty, integrity, respect, responsibility, and an understanding of others and different cultures [54]. In America, the values that are emphasized include generosity, helpfulness, honor, tolerance, talents, faith, kindness, honesty, justice, effective use of time, freedom of choice and expression, good citizenship, equality of opportunity, individual rights, and courage [55].

The values education curricula used in North Cyprus and Turkey are the same. The values promoted in the curriculum consist of tolerance, hospitality, patriotism, truthfulness, goodness, cleanliness, cooperation, solidarity, diligence, honesty, love, respect, sensitivity, fairness, and sharing. Schools need to establish a values-centred culture that emphasizes the teaching of these values. This responsibility should be shared among all education stakeholders [55]. To create a value-centered school culture, a six-stage planning process is necessary.The stages of values education are as follows:

1. Values-Based Vision and Strategic Planning: Establishing a clear vision that incorporates values into the planning process.
2. An Integrated Program: Developing a cohesive program that weaves values into all curricula, activities, and practices throughout the school.
3. Modeling Values: Ensuring that everyone in the school community, whether explicitly or implicitly, represents these values in all relationships and interactions.
4. Professional Development for Staff: Providing training and resources for staff to enhance their understanding and teaching of values education.
5. Collaboration with Stakeholders: Working together with school stakeholders to integrate values into implementation processes and all aspects of education.
6. Relevance to Society and School: Considering the needs of society, historical and cultural values, methods for interpreting values, and behavioral standards that arise from these values. This also includes shaping interpersonal relationships within the school and the broader community where values education is intended to be delivered [4].

Values education plays a foundational role in shaping individuals’ moral reasoning, ethical awareness, prosocial orientations, perceptions of fairness, and justice within organizations. By promoting values such as respect, responsibility, honesty, and fairness, values education helps individuals develop a strong sensitivity to ethical standards and equitable treatment [51,52]. When these values are reinforced within organizational settings—such as schools—they contribute to employees’ perceptions of organizational justice, including fairness in decision-making, interpersonal treatment, and outcomes. Organizational justice, in turn, serves as a critical mechanism through which values-based practices translate into positive employee behaviors. Drawing on Social Exchange Theory, fair and ethical treatment by an organization creates a sense of obligation among employees to reciprocate through discretionary behaviors that go beyond formal job requirements [41]. These discretionary actions are conceptualized as organizational citizenship behavior (OCB). Empirical research consistently demonstrates that employees who perceive high levels of organizational justice are more likely to engage in OCB, such as helping colleagues, showing commitment, and supporting organizational goals [42,56].

Thus, values education indirectly fosters organizational citizenship behavior by cultivating ethical orientations that enhance perceptions of organizational justice. When individuals internalize values emphasizing fairness and social responsibility, they are more likely to respond positively to just organizational practices and engage in citizenship behaviors that strengthen organizational effectiveness and sustainability [57].This interconnected relationship highlights values education as an important antecedent of organizational justice and OCB, particularly in educational organizations.

### Organizational citizenship

Organizational citizenship behaviors refer to actions taken by employees that, while not mandatory for their specific tasks or roles, contribute positively to the growth and operations of the organizations [58]. Almost all research on organizational citizenship behavior (OCB) is based on a social exchange perspective of citizenship performance [59]. Organizations gain advantages from employee contributions that exceed formal job requirements, known as Organizational Citizenship Behavior (OCB) [56]. These behaviors contribute to the overall effective functioning of the organization as a whole [60]. Organizational Citizenship Behaviors directed at individuals involve helping actions towards specific coworkers, such as assisting a sick colleague. In contrast, Organizational Citizenship Behaviors directed at the organization include actions that benefit the organization such as participation of an employee in a voluntary company fundraiser [61,62]. According to [63], there are five different types of organizational citizenship behavior that are altruism, courtesy, civic virtue, sportsmanship, and conscientiousness [64]. Altruism can be defined as a motivational state of increasing welfare of colleagues and peers in job-related tasks [65] such as dedicating time to help others without expecting anything in return. This support enhances individual performance and boots the efficiency of the groups [66]. Sportsmanship refers to ability to handle challenges without complaining [67], do not take offense easily, maintaining a positive outlook despite setbacks, handle rejection gracefully, prioritize collective interests over personal ones [57], and avoiding unnecessary grievances [66]. [68] highlight that a high level of sportsmanship creates a positive atmosphere, creates a harmonious work environment, and promotes collaboration which in turn helps improve productivity within an organization. Conscientiousness represents diligence and self-discipline. Research has shown it to be a strong predictor of academic success [69] and is the most significant predictor of problem-solving coping strategies in response to stressors [70]. Conscientiousness refers to behavior that goes beyond minimal expectations, such as effective time management and surpassing basic requirements. This quality improves both personal and group efficiency [66]. Courtesy is defined as polite and considerate behavior towards colleagues such as providing advance notices and reminders, which can help prevent complications and promote effective use of time [66]. Employees who demonstrate courtesy intentionally avoid creating problems for others, build positive relationships among colleagues, and contribute to a friendly and supportive work environment [71]. This helps to lessen the burden on management and enhance overall organizational performance [72], and prevents issues rather than just addressing them after they arise [73]. According to [74], civic virtue is associated with responsible behavior, which includes keeping up with organizational changes, suggesting improvements, and protecting the organization’s resources. Also, civic virtue includes behaviors that prioritize participation in significant organizational matters, such as voluntary attendance at events and committee work. It reflects an employee’s tendency to positively represent and promote their organization’s image [71]. All strengthen the interests of organizations [66].

Organizational Citizenship Behavior is widely recognized as a vital component of organizational development [75]. It has an important role in fostering a psycho-social work environment that enhances the organization’s fundamental activities [76]. Organizational Citizenship Behavior of employees has many positive effects in organizations [6]. For example OCB involves commitments that enhance organizational performance and increase productivity when accumulated among individuals over time [7]. Also, OCB of employees lowers their intention to leave [8].

### Organizational justice

Organizational justice is the perception of fairness within the workplace [77]. It encompasses how individuals or groups perceive their treatment by the organization, as well as their behavioral responses to these perceptions [78]. Additionally, these perceptions are related to organizational commitment and job satisfaction of employees [79]. It is accepted that the use, conceptualization and dissemination of the concept of organizational justice in general organization literature began with the work of [80]. Organizational justice can be understood through three distinct dimensions:

1. Distributive Justice: This refers to employees’ perceptions of how resources, rewards, and opportunities are allocated [81].
2. Procedural Justice: This dimension focuses on employees’ perceptions of fairness concerning the processes and decision-making involved in distributing resources [82].
3. Interactional Justice: This aspect addresses the quality of the relationships and interactions between employees within the organization [83].

Each dimension plays a critical role in shaping employees’ overall sense of justice within their workplace. Perceptions of interactional justice significantly predict future leaders’ willingness to support their subordinates, and their psychological well-being. Having interactional justice in work affects both organizations and employees [84]. Ensuring interactional justice in the workplace helps employees feel they are treated equally [85]. Management of an organization based on values affect organizational justice perception of employees. Management by values is an approach that recognizes that people can exhibit both rational and irrational behavior [86]. Management with keeping values focus on developing talent, motivating individuals, fostering innovation and synergy [87]. According to [88], management by values can foster an organizational culture that enables all employees to realize their personal and professional potential. Organizational justice is closely related to many organizational concepts. The main studies examining these relationships in the literature after the 2000s are: organizational culture [9], organizational citizenship behaviors [89–91], organizational trust [29,92], leadership [93–97], job satisfaction [92,98–103], organizational commitment [99], value systems [10], values [11], management according to values [12]. Decision-making processes, the nature of relationships, and resource distribution within the organization are related to the organizational justice perception of employees. When employees believe that managers do not ensure fairness, this lowers their commitment and performance [104], as well as trust and productivity, ultimately leading to higher employee turnover rates [105].

### Aim of the research

This research aimed to determine the mediating role of organizational justice in the relationship between teachers’ perceptions of value-centered school culture and their views on organizational citizenship. In addition, answers were sought to the following sub-problems of the study:

1. What are the levels of teachers’ perceptions regarding organizational justice, organizational citizenship, and value-centered school culture?
2. Is there a significant relationship between teachers’ perceptions of organizational justice, organizational citizenship, and value-centered school culture?
3. Do teachers’ perceptions of organizational justice predict their views on organizational citizenship?
4. Do teachers’ perceptions of a value-centered school culture predict their views on organizational citizenship?
5. Does organizational justice mediate the relationship between value-centered school culture and organizational citizenship among teachers?

## Method

### Research model

This study utilized a cross-sectional predictive correlational research design to explore the relationships between value-centered school culture, organizational justice, and organizational citizenship. In the proposed model, value-centered school culture is considered the predictor variable, organizational justice acts as the mediator variable, and organizational citizenship is identified as the outcome variable. Observed-variable path analysis was utilized to analyze both the direct and indirect relationships among these variables and to test the hypothesized mediation model. It is important to note that the data were collected at a single point in time using self-report measures, which means that the findings illustrate associations rather than causal relationships [106].

### Population and sample

The population of this study comprised 674 teachers employed at state secondary schools in the two largest cities of Northern Cyprus, Nicosia and Kyrenia, during the 2024–2025 academic year. These teachers were distributed among 18 state secondary schools. Nicosia and Kyrenia were chosen for this research due to their high concentration of public secondary schools and their representation of urban educational contexts in Northern Cyprus. The sampling frame was created using official teacher lists obtained from the Ministry of Education. A simple random sampling technique was used to ensure that every teacher in the population had an equal chance of being selected. This approach minimizes selection bias and enhances the representativeness of the sample. Before collecting data, the researchers obtained formal permissions from the school administrations. Teachers were reached through their respective schools. Participation in the study was voluntary, and participants were informed about the study’s purpose, the confidentiality of their responses, and their right to withdraw at any time without penalty. A total of 305 teachers completed the questionnaire. After data screening, four questionnaires were excluded due to missing or incomplete responses. As a result, the final valid sample consisted of 301 teachers. The study achieved a response rate of 45.4%, based on 305 out of 674 teachers initially contacted. Once the incomplete questionnaires were excluded, the final usable response rate was 44.7%, with 301 valid responses. This response rate is considered acceptable for survey-based research in educational settings and supports the adequacy of the sample size for the statistical analyses conducted. Participants’ personal characteristics are listed in Table 1.

**Table 1:** Participant teachers’ socio-demographic characteristics.

	Number (n)	Percentage (%)
**Gender**		
Female	208	69.10
Male	93	30.90
**Age**		
35 and below	91	30.20
36-46	118	39.20
47 and over	92	30.60
**Marital status**		
Single	107	35.50
Married	194	64.50
**Education level**		
Undergraduate	192	63.80
Post-graduate	109	36.20
**Professional experience**		
10 years and below	92	30.60
11-20	103	34.20
21 years and above	106	35.20
Total	301	100.00

In Nicosia and Kyrenia, most of the teachers are female and so that sample data constitutes a reflection of the population. According to Table 1, it was determined that most of the participants were female, married, had bachelor degree and longer years of experience in teaching.

### Data collection

Before data collection, research permission was obtained from the ethics committee of the researchers’ university (No: 2024/11.008 dated November 25, 2024). Permission was then obtained from the Ministry of National Education to administer the survey to teachers working in schools affiliated with the Ministry of National Education. All participants received a written consent form, which they read and signed. Data were collected through a survey conducted between December 2024 and March 2025 during the 2024–2025 academic year. A survey was developed by the researchers to collect data. The survey consisted of four parts: a personal information form, the Value-Centered School Culture Scale, the Organizational Justice Scale, and the Organizational Citizenship Scale. The personal information form included questions to obtain information about participants’ gender, age, marital status, education level, and length of professional experience.

Value-Centered School Culture Scale was developed by [4]. According to the results of the Exploratory Factor Analysis (EFA), the scale consists of 35 items divided into six sub-dimensions. Following a Confirmatory Factor Analysis (CFA), two additional items that disrupted the structure of the scale were removed, resulting in a refined 33-item scale. The analysis of the 33-item scale revealed a Chi-square fit test ratio (χ^2^/sd) of 2.52 (χ^2^/sd = 1204.949/477), indicating an excellent fit. The study found the following fit indices: Goodness of Fit Index (GFI) = 0.80, Comparative Fit Index (CFI) = 0.92, Root Mean Square Error of Approximation (RMSEA) = 0.071, Residual Mean Square (RMR) = 0.048, and Incremental Fit Index (IFI) = 0.92. These CFA results confirm that the model fit indices are at an acceptable level and that the factor structure of the scale is validated. Furthermore, the Cronbach’s Alpha reliability coefficients for the entire scale and its sub-dimensions were found to be very high:.964;.946;.915;.924;.807;.915; and.842, respectively. This data demonstrates that the scale exhibits high internal consistency. The final form of the Values-Centered School Culture Scale consists of 33 items organized into six dimensions. This scale uses a five-point Likert-type rating scale with the following options: “Strongly Disagree (1), Disagree (2), Undecided (3), Agree (4), Strongly Agree (5).” The dimensions of this scale include: monitoring and evaluation of values education, school-environment collaboration, values-based vision and strategic planning, teacher modeling, school administration-teacher collaboration, and staff professional development.

The Organizational Citizenship Behavior Scale for Schools is a 6-point Likert scale consisting of 12 items that assesses the extent to which a school’s teaching faculty engages in organizational citizenship behavior; a higher score indicates greater organizational citizenship within the school. It was developed by [66] and later translated into Turkish by [90]. This scale has a single dimension, where higher scores indicate a greater level of organizational citizenship behavior. The researchers performed factor analysis in order to define the construct validity of the scale. The factor loads of all of the items in the scale were above.30 and the scale had one factor. Eigenvalue of the factor was calculated as 5.48 and factor loads of the items varied between.31 and.82. The variance explained by the scale was 45.66%. The Cronbach Alpha reliability coefficient was found to be α = .87. Corrected Item-total correlations in the scale varied between.27 and.75.

The Organizational Justice Scale is a six-point Likert-type scale developed by [107] and later translated into Turkish by [90]. The scale consists of a single dimension with 10 items. A higher score on the scale indicates more positive perceptions of organizational justice. [4] reorganized the response options for the scale as follows: 1 – Strongly Disagree, 2 – Disagree, 3 – Moderately Agree, 4 – Agree, and 5 – Strongly Agree. Researchers performed principal components analysis to define the construct validity of the organizational justice scale and found the factor loads of all of the items above.30. Scale has one factor. Eigenvalue of the factor was calculated as 6.17. Factor loads of the items varied between.44 and.89. The variance explained by the scale was found as 61.74%. The Cronbach Alpha reliability coefficient was found to be α = .92. Total correlations of the items in the scale varied between.39 and.85. The total correlation of the items exceeded 0.20, which was the discrimination index for the items. The researchers indicated that these items were consistent with the entire scale.

### Data analysis

The data were analyzed using the IBM SPSS Statistics 28 package program, taking into account the purpose of the study and the questions. The data were considered to be normally distributed because Skewness values were between ‐2 and +2 and Kurtosis values were between ‐7 and +7 [108] so that parametric tests were performed to analyze the data. The mean, standard deviation, minimum, and maximum values of the scales were calculated. Pearson correlation analysis was conducted to explore the relationship between teachers’ perceptions of organizational justice, organizational citizenship, and value-centered school culture. Additionally, regression analysis was performed to assess the predictive power of teachers’ perceptions of organizational justice on their views about organizational citizenship, as well as the predictive power of teachers’ perceptions of a value-centered school culture on their views about organizational citizenship.

Observed-variable path analysis was conducted using observed composite variables to investigate the direct and indirect relationships among value-centered school culture, organizational justice, and organizational citizenship. This method is suitable for testing mediation models and allows for the simultaneous estimation of multiple relationships among the observed variables [109,110].

To evaluate the mediating role of organizational justice, a partial mediation model was developed. In this model:

- Value-centered school culture was treated as an exogenous variable,
- Organizational justice served as the mediator,
- Organizational citizenship was considered the endogenous outcome variable.

The analysis adhered to established mediation procedures [111].

## Findings

The findings of this research were analyzed in the light of the research questions.

As shown in Table 2, internal consistency reliability analyses were conducted for each measurement instrument and its corresponding sub-dimensions. According to widely accepted psychometric criteria, Cronbach’s alpha coefficients above 0.70 indicate acceptable internal consistency, values exceeding 0.80 suggest good reliability, and values above 0.90 represent excellent reliability. The analysis reveals that the reliability coefficients across all instruments range from a minimum of 0.851 to a maximum of 0.988, demonstrating high to excellent internal consistency. In particular, the Organizational Citizenship Behavior Scale (α = 0.933), the Organizational Justice Scale (α = 0.960), and the Value-Centered School Culture Scale (α = 0.941) all exceed the stringent 0.90 threshold for excellent instrument dependability. Furthermore, all five sub-dimensions of the Value-Centered School Culture Scale exhibited strong internal consistency, with coefficients ranging from 0.851 (Staff Professional Development) to 0.988 (Teacher Modeling). These results collectively indicate that the survey scales are highly reliable, stable, and statistically robust for subsequent multivariate inferential analyses.

**Table 2:** Reliability analysis of the scales.

Scale	Cronbach alpha value
Organizational Citizenship Behavior Scale	0.933
Organizational Justice Scale	0.960
Value-Centered School Culture Scale	0.941
Monitoring and evaluation of values education	0.945
School-environment collaboration	0.942
Values-based vision and strategic planning	0.957
Teacher modeling	0.988
School administration-teacher collaboration	0.952
Staff professional development	0.851

The Kolmogorov-Smirnov test results showed that organizational justice, organizational citizenship, and the dimensions of value-centered school culture scales had statistically significant values (p < .05), indicating deviations from a normal distribution.The Kolmogorov-Smirnov test is very sensitive to large sample sizes, which can cause the null hypothesis to be rejected even when the deviations from normality are minor (Ghasemi and Zahediasl, 2012). The Skewness values ranged from −0.616 to 0.484, and the Kurtosis values ranged from −0.778 to 0.282. These values fall within the acceptable thresholds of ±2, suggesting approximate normality (Hair et al., 2019). Therefore, the distributions were considered approximately normal, and the parametric statistical techniques were used to analyze the data (Table 3).

**Table 3:** Distribution of teachers’ Organizational Justice Scale, Organizational Citizenship Scale, and Value-Centered School Culture Scale scores.

	Kolmogorov-Smirnov				
	Statistics	sd	p	Skewness	Kurtosis
Organizational justice scale (Total)	0.076	301	p < .001	−0.352	−0.710
Organizational citizenship scale (Total)	0.068	301	0.002	−0.616	0.282
Sub-dimension 1: Monitoring and evaluation of values education	0.113	301	p < .001	0.179	−0.778
Sub-dimension 2: School-community collaboration	0.084	301	p < .001	0.282	−0.741
Sub-dimension 3: Values-based vision and strategic planning	0.073	301	0.001	−0.183	−0.764
Sub-dimension 4: Teacher modeling	0.088	301	p < .001	−0.176	0.055
Sub-dimension 5: School administration-teacher collaboration	0.166	301	p < .001	0.484	−0.503
Sub-dimension 6: Staff professional development	0.11	301	p < .001	−0.316	−0.037
Value-Centered School Culture Scale (Total of 6 sub-dimensions)	0.073	301	0.001	0.281	−0.685

**Research Question 1.** What are the levels of teachers’ perceptions regarding organizational justice, organizational citizenship, and value-centered school culture?

The participants received an average of 32.29 ± 10.12 points from the Organizational Justice Scale and 41.39 ± 10.38 points from the Organizational Citizenship Scale. The participants in the study received 15.14 ± 5.38 points from the Values education monitoring and evaluation sub-dimension of the Values-Centered School Culture Scale, 20.38 ± 7.70 points from the school-environment collaboration sub-dimension, 22.26 ± 7.49 points from the values-based vision-strategic planning sub-dimension, 22.57 ± 3.70 points from the teacher modeling sub-dimension, 13.63 ± 5.62 points from the school administration-teacher collaboration sub-dimension, and 9.93 ± 2.33 points from the staff professional development sub-dimension. The total score of the Values-Centered School Culture Scale was 103.91 ± 29.51 (Table 4).

**Table 4:** Teachers’ scores on the Organizational Justice Scale, Organizational Citizenship Scale, and Value-Centered School Culture Scale.

	n	$\overset{―}{x}$	s	Min	Max
Organizational Justice Scale(Total)	301	32.29	10.12	10	50
Organizational Citizenship Scale (Total)	301	41.39	10.38	12	59
Sub-dimension 1: Monitoring and Evaluating Values Education	301	15.14	5.38	5	25
Sub-dimension 2:School-Community Collaboration	301	20.38	7.70	7	35
Sub-dimension 3:Values-Based Vision-Strategic Planning	301	22.26	7.49	7	35
Sub-dimension 4:Teacher Modeling	301	22.57	3.70	12	30
Sub-dimension 5:School Administration-Teacher Collaboration	301	13.63	5.62	5	25
Sub-dimension 6: Staff Professional Development	301	9.93	2.33	3	15
Value-Centered School Culture Scale (Total of 6 sub-dimensions)	301	103.91	29.51	42	162

**Research Question 2.** Is there a significant relationship between teachers’ perceptions of organizational justice, organizational citizenship, and value-centered school culture?

Correlations between sub-dimensions and total scores of the three scales used in this study involve part-whole relationships. Statistically significant and positive correlations were found between the scores of teachers on the Organizational Justice Scale and Organizational Citizenship Scale (r = 0.676; p < 0.05) and between the scores of the teachers from the Organizational Justice Scale and the scores they received from the Value-Centered School Culture Scale (r = 0.658; p < 0.05). The results of the data analysis showed significant and positive correlations between organizational justice and all sub-dimensions of value-centered school culture ranging from r = .436 to r = .724. In addition, significant and positive correlations were found between the organizational citizenship scale scores and sub-dimensions of value-centered school culture scores ranging from r = .440 to r = .753 (Table 5).

**Table 5:** Correlations between teachers’ organizational justice, organizational citizenship, and Value-centered School Culture Scale scores.

		1	2	3	4	5	6	7	8	9
**Organizational Justice Scale(1)** **Orga**nizational Citizenship Scale	r	1								
	p									
	N	301								
**Organizational Citizenship Scale(2)**	r	.676**	1							
	p	p < .001								
	N	301	301							
Monitoring and Evaluating Values Education(3)	r	.540**	.522**	1						
	p	p < .001	p < .001							
	N	301	301	301						
School-Community Collaboration(4)	r	.635**	.621**	.947**	1					
	p	p < .001	p < .001	p < .001						
	N	301	301	301	301					
Values-BasedVision-Strategic Planning,Teacher Modeling (5)	r	.724**	.753**	.711**	.828**	1				
	p	p < .001	p < .001	p < .001	p < .001					
	N	301	301	301	301	301				
Values-Based Vision-Strategic Planning (6)	r	.436**	.440**	.751**	.763**	.597**	1			
	p	p < .001	p < .001	p < .001	p < .001	p < .001				
	N	301	301	301	301	301	301			
School Administration-Teacher Collaboration(7) Staff Professional Development	r	.555**	.532**	.942**	.956**	.700**	.764**	1		
	p	p < .001	p < .001	p < .001	p < .001	p < .001	p < .001			
	N	301	301	301	301	301	301	301		
School Administration-Teacher Collaboration (8)	r	.626**	.596**	.770**	.823**	.790**	.690**	.737**	1	
	p	p < .001	p < .001	p < .001	p < .001	p < .001	p < .001	p < .001		
	N	301	301	301	301	301	301	301	301	
**Value-Centered School Culture Scale (9)**	r	.658**	.652**	.944**	.986**	.870**	.813**	.943**	.862**	1
	p	p < .001	p < .001	p < .001	p < .001	p < .001	p < .001	p < .001	p < .001	
	N	301	301	301	301	301	301	301	301	301

**Research Question 3.** Do teachers’ perceptions of organizational justice predict their views on organizational citizenship?

**Fig 1:**
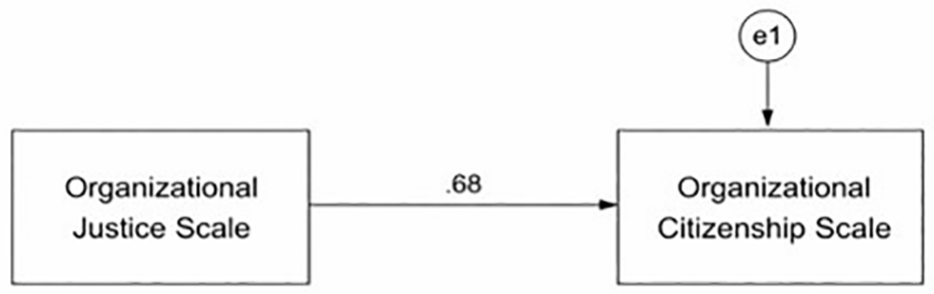
Prediction of teachers’ Organizational Justice Scale scores on Organizational Citizenship Scale scores.

Path analysis was used to examine the predictive value of the teachers’ Organizational Justice Scale scores on the Organizational Citizenship Scale scores, as shown in Fig 1. It was determined that the teachers’ Organizational Justice Scale scores predicted the Organizational Citizenship Scale scores statistically significantly and positively (β = 0.68; p < 0.05) (Fig 1).

**Research Question 4.** Do teachers’ perceptions of a value-centered school culture predict their views on organizational citizenship?

**Fig 2:**
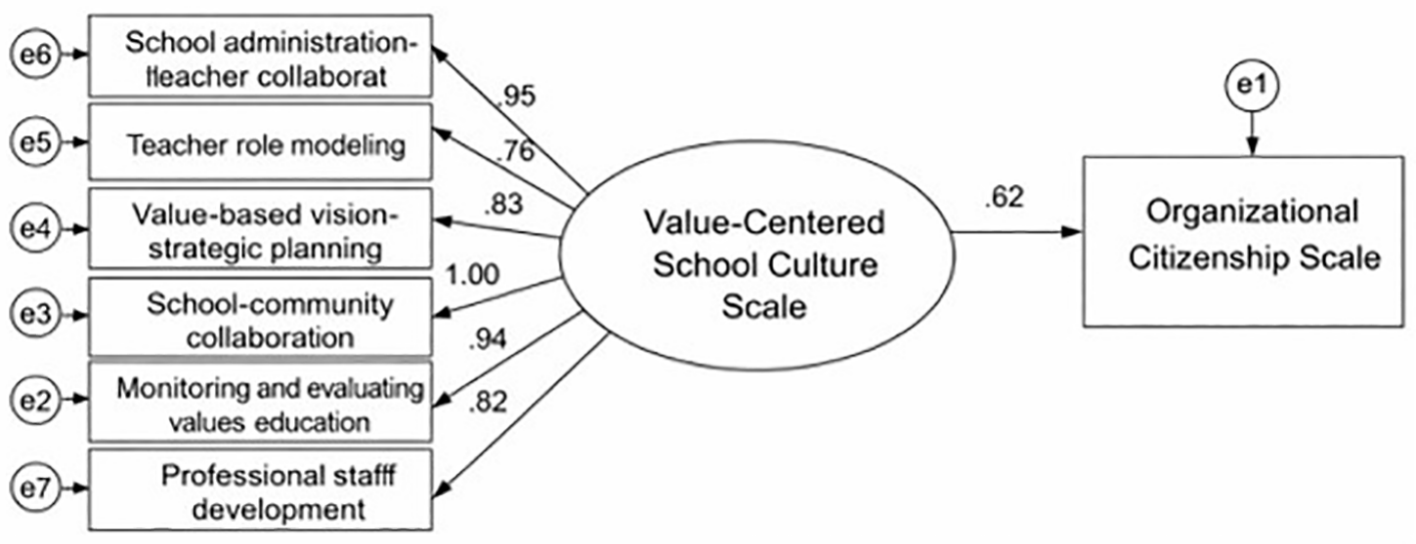
Prediction of teachers’ Value-centered School Culture Scale scores on Organizational Citizenship Scale scores.

Structural equation modeling (SEM) treating Value-Centered School Culture as a latent construct with six dimensions was utilized to address research question 4 and to account for measurement error among the subscales. It was determined that teachers’ Value-Centered School Culture Scale scores predicted Organizational Citizenship Scale scores statistically significantly and positively (β = 0.62; p < 0.05) (Fig 2).

The adequacy of the proposed latent variable structural model was assessed using multiple goodness-of-fit indices. The model demonstrated good to excellent fit indices (χ^2^ = 21.518; df = 14; p = 0.089; **χ^2^/sd =** 1.537; GFI = 0.910; NFI = 0.911; CFI = 0.916; RMSEA = 0.022), supporting the structural validity of the framework [112,113] (Table 6).

**Table 6:** Goodness of fit indices for the latent variable structural model (Fig 2).

Index	Value	Limit value
**χ^2^/sd**	1.537	≤ 3
**GFI**	0.910	0.90−0.95
**NFI**	0.911	0.90−0.95
**CFI**	0.916	0.90−0.95
**RMSEA**	0.022	≤ 0.05

Research Question 5. Does organizational justice mediate the relationship between value-centered school culture and organizational citizenship among teachers?

**Fig 3:**
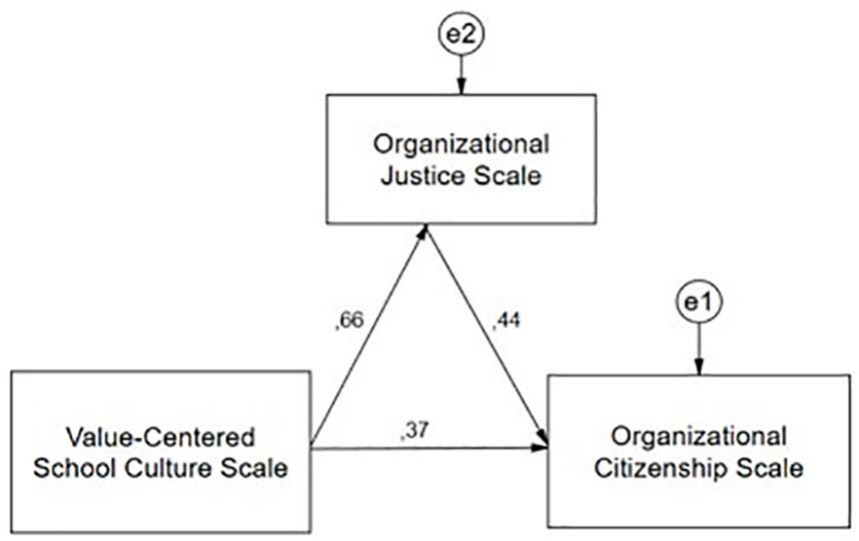
The mediating role of the Organizational Justice Scale in the relationship between teachers’ Value-Centered School Culture Scale scores and Organizational Citizenship Scale scores.

Path analysis including observed composite scores was conducted to evaluate the specific mediation paths and direct/indirect effects via bootstrapping. Fig 3 shows that Value-Centered School Culture Scale scores have a statistically significant and positive effect on Organizational Justice Scale scores (β = 0.66; p < 0.001). Furthermore, Organizational Justice Scale scores also have a statistically significant and positive effect on Organizational Citizenship Scale scores (β = 0.44; p < 0.001). In addition, a significant direct effect of Value-Centered School Culture Scale scores on Organizational Citizenship Scale scores was found (β = 0.37; p < 0.001). In the mediation analysis conducted using the Bootstrap method with 5.000 resamplings, the indirect effect of the Value-Centered School Culture Scale on the Organizational Citizenship Scale was found to be significant (B = 0.101; Bootstrap SE = 0.016; 95% Bootstrap CI = 0.070–0.133). The absence of a zero confidence interval indicates that the indirect effect is statistically significant. Since the direct effect also retained its significance, it was concluded that the Organizational Justice Scale plays a partial mediating role in the relationship between Value-Centered School Culture and Organizational Citizenship.

## Discussion and conclusion

This quantitative research aimed to determine the mediating role of organizational justice in the relationship between teachers’ perceptions of value-centered school culture and their views on organizational citizenship. The results indicate that teachers generally experience a positive, value-driven school environment, perceive fairness in institutional processes, and exhibit citizenship behaviors. These findings are consistent with international research, which suggests that a supportive organizational climate is associated with discretionary behaviors among teachers. This indicates that the quality of a school’s internal environment plays a significant role in shaping teachers’ extra-role behaviors beyond their formal duties [114].

The observed positive relationship between a value-centered culture and organizational justice is consistent with existing literature, which suggests that shared ethical principles and values within organizations are associated with stronger perceptions of fairness. For example, research conducted in private school settings has shown that when teachers perceive fairness in areas such as resource distribution, decision-making, and interpersonal relationships, their levels of commitment and citizenship behaviors tend to increase [115]. This relationship is consistent with Social Exchange Theory: when organizational values are consistently upheld, teachers are more likely to perceive their work environment as equitable, which is associated with reciprocal behaviors such as organizational citizenship behavior (OCB). Supporting this [35], demonstrated that cultural value orientations can shape the relationship between justice perceptions and OCB across different countries, emphasizing the global relevance of these associations.

The association between organizational justice and OCB is supported by extensive cross-cultural evidence. Research shows that teachers who perceive procedural and interactional fairness are more likely to engage in extra-role behaviors that benefit their schools. Perceptions of fairness are also associated with higher levels of trust and job satisfaction, both of which are important factors related to organizational citizenship behavior in workplaces around the world [62]. In educational settings, this pattern has been observed across various regions, including Asia, Europe, and Africa, suggesting that organizational justice is consistently related to citizenship behaviors among teachers [116].

The link between a value-centered culture and organizational citizenship behavior (OCB) extends existing research by suggesting that a focus on ethical values is associated with teachers’ willingness to exceed their formal responsibilities. While previous studies have typically concentrated on organizational culture or school climate, this research introduces a more specific perspective by focusing on value-centered school culture, which emphasizes moral and social values. This concept closely aligns with educational policy objectives and reflects normative expectations in education systems that prioritize ethical behavior and character development.

The findings suggest that a value-centered school culture is positively associated with both organizational justice and organizational citizenship. Additionally, organizational justice is positively associated with organizational citizenship. Notably, when organizational justice was included in the model, the direct association between value-centered school culture and organizational citizenship decreased but remained significant, suggesting a partial mediating effect. The positive association between a value-centered school culture and organizational justice indicates that schools that prioritize shared values, ethics, and fairness tend to foster stronger perceptions of justice among teachers. This finding is consistent with previous research showing that organizational environments rooted in ethical values and transparency are associated with improved perceptions of fairness [104,105].

The substantial association between organizational justice and organizational citizenship behaviors aligns with existing literature. Teachers who perceive fairness in decision-making processes, interpersonal treatment, and resource distribution are more likely to engage in discretionary behaviors that exceed their formal job requirements [57,63]. This observation supports Social Exchange Theory, which proposes that employees tend to reciprocate fair treatment with positive workplace behaviors.

The finding that a value-centered school culture is associated with organizational citizenship behaviors highlights the importance of cultural values in encouraging voluntary and cooperative actions among teachers. Schools that promote respect, collaboration, and a shared moral purpose may foster a stronger sense of belonging and commitment among educators, which is in turn associated with higher levels of organizational citizenship behavior [46].

A key finding is that organizational justice plays a partial mediating role, indicating that a value-centered school culture is associated with organizational citizenship both directly and indirectly through perceptions of fairness. This suggests that while a strong value-based culture may independently relate to positive behaviors, its association is strengthened when teachers perceive the organization as fair. Similar mediation patterns have been reported in previous studies highlighting organizational justice as a mechanism linking organizational climate and employee behaviors [117]. The observed-variable path analysis indicated a good to excellent fit, supporting the adequacy of the proposed relationships and the overall model structure.

Overall, these findings emphasize the importance of developing both a value-centered school culture and a fair organizational environment in relation to teachers’ organizational citizenship behaviors. Therefore, educational leaders are encouraged to prioritize ethical practices, transparency, and equitable decision-making processes alongside the development of a value-based culture.

### Limitations

This study is valuable, but it has several limitations that should be considered when interpreting its findings. First, the research used a cross-sectional relational design, which limits the ability to draw definitive causal conclusions about value-centered school culture, organizational justice, and organizational citizenship. Although observed-variable path analysis was employed to examine predictive relationships, the results reflect associations rather than causal effects. Second, the data were collected exclusively using self-report questionnaires from a single source (teachers). This approach may introduce common method bias and social desirability effects, as participants’ responses may reflect subjective perceptions rather than objective organizational conditions or behaviors. The use of a single-source design may therefore inflate the observed relationships among variables.

Third, the sample included only teachers from general secondary schools in two major cities in North Cyprus. As a result, the findings may not be generalized to other educational levels, geographical regions, or cultural contexts. Differences in educational systems and organizational cultures may predict teachers’ perceptions of justice and citizenship behaviors.

Fourth, the analysis did not include within-sample confirmatory factor analysis (CFA) to revalidate the measurement model in the current dataset. Although the scales used in this study have been validated in previous research, the absence of a within-sample CFA limits the ability to confirm construct validity in the present context. Future research should incorporate CFA to further strengthen measurement validation.

Another important limitation relates to the high inter correlations observed among some sub-dimensions of the Value-Centered School Culture Scale. Several correlations exceeded commonly accepted thresholds for discriminant validity, suggesting potential conceptual overlap among certain dimensions. This indicates that participants may have perceived these sub-dimensions as closely related aspects of a broader construct rather than fully distinct factors. While the scale has demonstrated validity in prior studies, these findings suggest that further psychometric evaluation of discriminant validity is needed across different samples.

### Recommendations

Based on the findings of this study, several recommendations for future research are proposed. First, future studies should use longitudinal or experimental research designs to better investigate the causal relationships among values-centered school culture, organizational justice, and organizational citizenship. Longitudinal data will enable researchers to observe changes over time and provide stronger evidence concerning the directionality of these relationships. Second, it is recommended that future research use mixed-methods approaches that combine quantitative surveys with qualitative techniques like interviews, focus groups, or observations. Qualitative data can provide deeper insights into how a values-centered school culture is implemented in schools and how teachers perceive organizational justice and citizenship behaviors in their daily professional experiences. Third, to improve the generalizability of the findings, it is important to expand the sample to include teachers from various educational levels, such as primary and higher education, as well as from different geographic regions and cultural contexts. Conducting cross-cultural and comparative studies could be particularly valuable in determining whether the observed relationships are consistent across diverse educational systems. Furthermore, future research should involve multiple stakeholders, including school administrators, students, and support staff, to provide a more comprehensive understanding of organizational culture and justice within schools

Examining leadership roles, such as ethical or transformational leadership, can help clarify how a values-centered school culture is developed and maintained. Additionally, future research should consider controlling for or investigating various organizational and individual factors, such as leadership style, school climate, workload, job stress, teacher tenure, and institutional policies. Finally, employing multi-level modeling techniques in future studies could enhance our understanding by capturing both school-level and individual-level relations. This approach would allow researchers to explore how organizational characteristics interact with individual perceptions and behaviors, leading to a more nuanced understanding of organizational dynamics in educational settings.

## References

[1] Peters TJ, Waterman RH. In Search of Excellence: Lessons from America’s Best-Run Companies. New York: Harper & Row; 1982.

[2] Robbins SP, Judge TA. Organisational Behavior. 15th ed. Boston: Pearson; 2013.

[3] Aktepe V, Keser H, Şeref ŞE. Evaluation of values and values education practices from the perspective of classroom teachers. Afyon Kocatepe Univ J Soc Sci. 2020;22(4):897–918. doi: 10.32709/akusosbil.781857

[4] Zengin M, Çelik ME. Development of the value-centered school culture scale: validity and reliability study. J Values Educ. 2019;17(37):317–48. doi: 10.34234/ded.541321

[5] Sezgin F. Organizational citizenship behaviors: a conceptual analysis and some implications from a school perspective. Gazi Univ J Gazi Faculty Educ. 2005;25(1):317–39.

[6] Vagner B, Blix L, H H, Ortegren M, Sorensen K. Upward feedback falling on deaf ears: the effect on provider organizational citizenship and counterproductive work behaviors in the audit profession. Manag Audit J. 2022;37:17–38. doi: 10.1108/MAJ-09-2020-2845

[7] Guntuku RK, Nanak G, Teknik I. Organizational citizenship behavior at the workplace: contributions to institutional excellence. South Asia J Bus Manage Res. 2020;10:11–21. doi: 10.5958/2249-877X.202p≤.00110.7

[8] Abror A, Patrisia D, Syahrizal S, Sarianti R, Dastgir S. Self-efficacy, employee engagement, remuneration and employee loyalty in higher education: the role of satisfaction and OCB. Int J Adv Sci Technol. 2020;29(3):5456–70.

[9] Beugré CD. Understanding organizational justice and its impact on managing employees: An African perspective. Int J Hum Resour Manage. 2002;13(7):1091–104. doi: 10.1080/09585190210131311

[10] Lipponen J, Olkkonen M-E, Myyry L. Personal value orientation as a moderator in the relationships between perceived organizational justice and its hypothesized consequences. Soc Justice Res. 2004;17(3):275–92. doi: 10.1023/b:sore.0000041294.68845.0f

[11] Fischer R, Smith PB. Values and organizational justice: performance-and seniority-based allocation criteria in the United Kingdom and Germany. J Cross-Cult Psychol. 2004;35(6):669–88. doi: 10.1177/0022022104270110

[12] Altınkurt Y, Yılmaz K. Examining the relationship between values-based management and organizational justice according to secondary school teachers’ perceptions. Educ Admin: Theory Pract. 2010;16(4):463–84.

[13] Arlı D. An examination of teachers’ organizational citizenship behaviors in terms of organizational culture perceptions and organizational trust levels. İzmir, Turkey: Ege University, Graduate School of Social Sciences; 2011.

[14] Ebrahimpour H, Zahed A, Khaleghkhah A, Sepehri MB. A survey relation between organizational culture and organizational citizenship behavior. Procedia - Soc Behav Sci. 2011;30:1920–5. doi: 10.1016/j.sbspro.2011.10.373

[15] İpek C. Organizational culture and organizational citizenship behavior in secondary education institutions according to teachers’ perceptions. Educ Admin: Theory Pract. 2012;18(3):399–434.

[16] Mohanty J, Rath NBP. Influence of organizational culture on organizational citizenship behavior: a three-sector study. Glob J Bus Res. 2012;6(1):65–76.

[17] Koşar D, Yalçınkaya M. Organizational culture and organizational trust as predictors of teachers’ organizational citizenship behaviors. Educ Admin: Theory Pract. 2013;19(4):603–27.

[18] Avcı A. The effect of organizational culture on organizational citizenship behaviors. J Hum Sci. 2016;13(3):5373–98. doi: 10.14687/jhs.v13i3.4264

[19] Sökmen A, Benk O, Gayaker S. The relationship between organizational culture, organizational citizenship behavior, and organizational commitment: a study in a public institution. Gazi Univ J Faculty Econ Admin Sci. 2017;19(2):415–29.

[20] Erdogan B, Liden RC. Collectivism as a moderator of responses to organizational justice: implications for leader‐member exchange and ingratiation. J Organ Behavior. 2006;27(1):1–17. doi: 10.1002/job.365

[21] Cropanzano RS, Ambrose ML. Culture and organizational justice: State of the literature and suggestions for future directions. Cropanzano RS, Ambrose ML, The Oxford handbook of justice in the workplace. Oxford University Press; 2015. pp. 273–90.

[22] Kim S, Tam L, Kim J, Rhee Y. Determinants of employee turnover intention: Understanding the roles of organizational justice, supervisory justice, authoritarian organizational culture and organization-employee relationship quality. Corp Communs: Int J. 2017;22(3):308–28.

[23] Najafi H, KHaleghkhah A. The prediction of perceived organizational justice of teachers based on the components of organizational culture: a new strategy for improving school management. School Admin. 2019;7(2):148. doi: 10.34785/J010.2019.785

[24] Suhardi M, Huda SA, Mulyadi D, Nazopah N. The effect of organizational culture, leader behaviors, job satisfaction, and justice on organizational commitment. J Appl Sci Eng Technol Educ. 2020;2(1):37–42. doi: 10.35877/454ri.asci2147

[25] Gelfand MJ, Erez M. Reconsidering assumptions about organizational justice through the lens of culture and moral philosophy. The Oxford handbook of cross-cultural organizational behavior. Oxford University Press; 2024. pp. 9.

[26] Hermawan A, Wardani AK, Satriyo B. Enhancing the quality of teacher services through strengthening personality and organizational justice. Int J Multidiscip Res Growth Eval. 2025;6(1):397–406. doi: 10.54660/.IJMRGE.2025.6.1.397-406

[27] Atalay İ. Organizational citizenship and organizational justice. Afyon, Turkey: Afyon Kocatepe University, Graduate School of Social Sciences; 2005.

[28] Moorman RH, Byrne ZS. How does organizational justice affect organizational citizenship behavior?. In: Greenberg J, Colquitt JA, editors. Handbook of Organizational Justice. New Jersey. 2005. pp. 355–79.

[29] Polat S, Celep C. Organizational culture and teachers’ perceptions of organizational justice, organizational trust, and organizational citizenship behaviors. Educ Admin: Theory Pract. 2008;54(54):307–31.

[30] Goudarzvand-Chegini M. The relationship between organizational justice and organizational citizenship behavior. Am J Econ Bus Adm. 2009;1(2):171–4. doi: 10.3844/ajebasp.2009.173.176

[31] Guangling W. The study on relationship between employees’ sense of organizational justice and organizational citizenship behavior in private enterprises. Energy Procedia. 2011;5:2030–4. doi: 10.1016/j.egypro.2011.03.350

[32] Nghi AM. The study on relationship between employees’ sense of organizational justice and organizational citizenship behavior in private enterprises. Energy Procedia. 2011;5:2030–4. doi: 10.1016/j.egypro.2011.03.350

[33] Akyüz B. The impact of servant leadership behaviors on organizational justice, organizational citizenship behaviors, and performance: A study on the education sector. Gebze (TR): Gebze Institute of Advanced Technology; 2012.

[34] Jafari P, Bidarian S. The relationship between organizational justice and organizational citizenship behavior. Procedia - Soc Behav Sci. 2012;47:1815–20. doi: 10.1016/j.sbspro.2012.06.905

[35] Schilpzand MC, Martins LL, Kirkman BL, Lowe KB, Chen ZX. The relationship between organizational justice and organizational citizenship behaviour: The role of cultural value orientations. Manag Organ Rev. 2013;9(2):345–74. doi: 10.1111/more.12014

[36] Bahrami MA, Montazeralfaraj R, Gazar SH, Tafti AD. Relationship between organizational perceived justice and organizational citizenship behavior among an iranian hospital’s employees, 2013. Electron Phys. 2014;6(2):838–44. doi: 10.14661/2014.838-844 25763156 PMC4324267

[37] Yadav LK, Gupta P. Procedural justice, job satisfaction and organizational citizenship behaviour: mediating role of organizational trust—indian tourism industry study. Manage Labour Stud. 2017;42(3):275–92. doi: 10.1177/0258042x17718738

[38] Samancı S, Basım HN. Academics’ perceptions of organizational justice and organizational citizenship behaviors: the mediating role of psychological capital. Bus Econ Res J. 2018;9(2):363–80. doi: 10.20409/berj.2018.110

[39] Erkutlu H. The moderating role of organizational culture in the relationship between organizational justice and organizational citizenship behaviors. Leadersh Organ Dev J. 2011;32(6):532–54. doi: 10.1108/01437731111161058

[40] Uysal Ş, Sarıer Y, Çilek A. The factors affecting organizational citizenship behavior of teachers in Turkey: A meta-analysis study. J Contemp Manag Sci. 2019;6(2):183–202.

[41] Blau P. Power and exchange in social life. New York (NY): John Wiley & Sons; 1964.

[42] Chen HF. The relationships of organizational justice, social exchange, psychological contract, and expatriate adjustment: An example of Taiwanese business expatriates. Int J Hum Resour Manag. 2010;21:1090–107. doi: 10.1080/09585191003783520

[43] Prysmakova P. Institutional antecedents of public service motivation: administrative regime and sector of economy. Nonprofit Manag Leadersh. 2021;32(1):99–119. doi: 10.1002/nml.21464

[44] Kim J. The contrary effects of intrinsic and extrinsic motivations on burnout and turnover intention in the public sector. Int J Manpow. 2018;39:486–500. doi: 10.1108/IJM-03-2017-0053

[45] Michel JS, Hargis MB. What motivates deviant behavior in the workplace? An examination of the mechanisms by which procedural injustice affects deviance. Motiv Emot. 2017;41:51–68. doi: 10.1007/s11031-016-9584-4

[46] Deal TE, Peterson KD. Shaping school culture: The heart of leadership. San Francisco (CA): Jossey-Bass; 1999.

[47] Schoen LT, Teddlie C. A new model of school culture: a response to a call for conceptual clarity. Sch Eff Sch Improv. 2008;19(2):129–53. doi: 10.1080/09243450802095278

[48] Lee M, Louis KS. Mapping a strong school culture and linking it to sustainable school improvement. Teach Teach Educ. 2019;81:84–96. doi: 10.1016/j.tate.2019.02.001

[49] Kilag OK, Tokong C, Enriquez B, Deiparine J, Purisima R, Zamora M. School leaders: The extent of management empowerment and its impact on teacher and school effectiveness. Excell Int Multidiscip J Educ. 2023;1(1):127–40.

[50] Gruenert S, Whitaker T. School culture rewired: How to define, assess, and transform it. Alexandria (VA): ASCD; 2015.

[51] Halstead JM, Taylor MJ. Learning and teaching about values: a review of recent research. Camb J Educ. 2000;30(2):169–202. doi: 10.1080/03057640050075169

[52] Lovat T, Toomey R. Values education and quality teaching: The double helix effect. Dordrecht (NL): Springer; 2009.

[53] Toker Gökçe A. Core values in education from the perspective of future educators. Sage Open. 2021;11(2). doi: 10.1177/21582440211014485

[54] Snook I. National Framework for Values Education in Australian Schools. 2007. https://asmre.org/801/11/AustralianNatioalValuesFramework-2005.pdf

[55] Roth WM, Lee S. Science education as/for participation in the community. Sci Educ. 2004;88(2):263–91. doi: 10.1002/sce.10113

[56] Organ DW. Organizational citizenship behavior: recent trends and developments. Annu Rev Organ Psychol Organ Behav. 2018;5:295–306. doi: 10.1146/annurev-orgpsych-032117-104536

[57] Podsakoff PM, MacKenzie SB, Paine JB, Bachrach DG. Organizational citizenship behaviors: a critical review of the theoretical and empirical literature and suggestions for future research. J Manag. 2000;26:513–63. doi: 10.1177/014920630002600307

[58] Al-Ahmadi AT, Mahran SM. Organizational citizenship behavior and job satisfaction from the nurses’ perspective. Evid Based Nurs Res. 2022;4(1):9. doi: 10.47104/ebnrojs3.v4i1.230

[59] Zellars KL, Tepper BJ. Beyond social exchange: new directions for organizational citizenship behavior theory and research. Res Pers Hum Resour Manag. 2003;22:395–424. doi: 10.1016/S0742-7301(03)22009-0

[60] Fein EC, Tziner A, Vasiliu C. Perceptions of ethical climate and organizational justice as antecedents to employee performance: the mediating role of employees’ attributions of leader effectiveness. Eur Manag J. 2023;41:114–24. doi: 10.1016/j.emj.2021.11.003

[61] El-Kassar AN, Dagher GK, Lythreatis S, Azakir M. Antecedents and consequences of knowledge hiding: the roles of HR practices, organizational support for creativity, creativity, innovative work behavior, and task performance. J Bus Res. 2022;140:1–10. doi: 10.1016/j.jbusres.2021.11.079

[62] Rahman MHA, Karim DN. Organizational justice and organizational citizenship behavior: the mediating role of work engagement. Heliyon. 2022;8(5):e09450. doi: 10.1016/j.heliyon.2022.e09450 35620633 PMC9126923

[63] Organ DW. Organizational citizenship behavior: The good soldier syndrome. Lexington (MA): Lexington Books; 1988.

[64] Atatsi EA, Stoffers J, Kil A. Work tenure and organizational citizenship behaviors. Sustainability. 2021;13:1–14. doi: 10.3390/su132413762

[65] Ma E, Qu H, Wei X, Hsiao A. Conceptualization and operationalization of an altruistic and egoistic continuum of organizational citizenship behavior motivations. J Hosp Tour Res. 2018;42:740–71. doi: 10.1177/1096348015619412

[66] DiPaola MF, Hoy WK. Organizational citizenship of faculty and achievement of high school students. High Sch J. 2005;88(3):35–44. doi: 10.1353/hsj.2005.0002

[67] Lan J. Past, present or future?: The effects of temporal focus on employees’ discretionary behaviors. Hong Kong (HK): Hong Kong Baptist University; 2018.

[68] Puspitasari V, Hidayati T, Rahmawati R. Analyzing the effect of sportsmanship and civic virtue behaviors on teacher performance: moderating role of affective commitment. J Madani Soc. 2023;2(1):9–16. doi: 10.56225/jmsc.v2i1.173

[69] Icekson T, Kaplan O, Slobodin O. Does optimism predict academic performance? Exploring the moderating roles of conscientiousness and gender. Stud High Educ. 2020;45:635–47. doi: 10.1080/03075079.2018.1564257

[70] Abbas M, Raja U. Challenge-hindrance stressors and job outcomes: The moderating role of conscientiousness. J Bus Psychol. 2019;34(2):189–201. doi: 10.1007/s10869-018-9535-z

[71] Oamen TE. The impact of firm based organizational citizenship behavior on continuance and normative commitment among pharmaceutical executives: an SEM approach. J Econ Manage. 2023;45:47–67. doi: 10.22367/jem.2023.45.04

[72] Faajir A, Asenge EL, Ikyanyon DD. Effect of altruism and courtesy on the growth of listed deposit money banks (DMBs) in Nigeria. J Entrepreneurship Innov. 2021;1:79–92.

[73] Magdalena SM. The effects of organizational citizenship behavior in the academic environment. Procedia - Soc Behav Sci. 2014;127:738–42. doi: 10.1016/j.sbspro.2014.03.346

[74] Robbins SP, Judge TA. Organizational behavior. 16th ed. Harlow (UK): Pearson Education; 2015.

[75] Somech A, Ohayon BE. The trickle-down effect of OCB in schools: the link between leader OCB and team OCB. J Educ Adm. 2019;58:629–43. doi: 10.1108/JEA-03-2019-0056

[76] Organ DW, Ryan K. A meta-analytic review of attitudinal and dispositional predictors of organizational citizenship behavior. Pers Psychol. 1995;48:775–802. doi: 10.1111/j.1744-6570.1995.tb01781.x

[77] Cropanzano R, Byrne ZS, Bobocel DR, Rupp DE. Moral virtues, fairness heuristics, social entities, and other denizens of organizational justice. J Vocat Behav. 2001;58(2):164–209. doi: 10.1006/jvbe.2000.1750

[78] Lam SSK, Schaubroeck J, Aryee S. Relationship between organizational justice and employee work outcomes: a cross-national study. J Organ Behav. 2002;23(1):1–18. doi: 10.1002/job.131

[79] Günaydın SC. A study examining the effects of organizational justice and organizational trust on perceived political behavior and the tendency to cooperate in businesses. Istanbul (TR): Marmara University; 2001.

[80] Greenberg J. A taxonomy of organizational justice theories. Acad Manag Rev. 1987;12(1):9–22. doi: 10.5465/amr.1987.4306437

[81] Olsaretti S. Introduction: The idea of distributive justice. Oxford Handbook of Distributive Justice. Oxford (UK): Oxford University Press; 2018. doi: 10.1093/oxfordhb/9780199645121.013.38

[82] Yılmaz K. Teachers’ perceptions of organizational justice in public secondary schools. Educ Sci Theory Pract. 2010;10(1):579–616. doi: 10.12738/estp.2010.1.123

[83] Cohen-Charash Y, Spector PE. The role of justice in organizations: a meta-analysis. Organ Behav Hum Decis Process. 2001;86(2):278–321. doi: 10.1006/obhd.2001.2958

[84] Levenda AM, Behrsin I, Disano F. Renewable energy for whom? A global systematic review of the environmental justice implications of renewable energy technologies. Energy Res Soc Sci. 2021;71:101837. doi: 10.1016/j.erss.2020.101837

[85] Qandeel MS, Kurath G. A systematic review and meta-analysis: leadership and interactional justice. Manag Rev Q. 2023;75(1):1–30. doi: 10.1007/s11301-023-00384-y

[86] Çelik V. Educational leadership. Ankara (TR): Pegem A Yayıncılık; 2000.

[87] Institute of Value Management. Value management. London (UK): Institute of Value Management; 2001.

[88] García-Izquierdo AL, Moscoso S, Ramos-Villagrasa PJ. Reactions to the fairness of promotion methods: procedural justice and job satisfaction. Int J Sel Assess. 2012;20(4):394–403. doi: 10.1111/ijsa.12002

[89] Koopmann R Jr. The relationship between perceived organizational justice and organizational citizenship behaviors: A review of literature. Univ Wis Stout J Stud Res. 2002;1:137–44.

[90] Yılmaz K, Taşdan M. Organizational citizenship and organizational justice in Turkish primary schools. J Educ Adm. 2009;47(1):108–26. doi: 10.1108/09578230910930925

[91] Karriker JH, Williams ML. Organizational justice and organizational citizenship behavior: a mediated multifoci model. J Manag. 2009;35(1):112–35. doi: 10.1177/0149206307308918

[92] Yılmaz K, Karaköse T, Altınkurt Y. The relationship between organizational trust and organizational justice in primary schools. In: Proc 5th Int Balkan Educ Sci Congr. Edirne (TR): Trakya University Faculty of Education; 2009. pp. 319–322. doi: 10.1108/09578230910928106

[93] Kinter O, Seymen O. The relationship between transformational leadership style and organizational justice: A conceptual evaluation. Balıkesir Univ J Faculty Econ Admin Sci. 2020;1(2):31–53.

[94] Beycioğlu K, Şişman M. School principals’ leadership styles and perceptions of organizational justice. Educ Adm Theory Pract. 2020;26(1):1–24.

[95] Yeni G. The impact of 21st-century skills on school principals’ leadership styles. Int J Leadersh Educ. 2020;2(2):19–30.

[96] Bello EO, Oredein AO. School climate, principal managerial styles and organisational commitment among junior secondary school teachers in Oyo State, Nigeria. Int J Res. 2022;10(7):51–69. doi: 10.29121/granthaalayah.v10.i7.2022.4686

[97] Saima M, Idris M, Minaz M. The transformative leadership style of 21st century secondary school principals and its influence on school environment. Dialogue Soc Sci Rev. 2025;3(1):43–54.

[98] Clay-Warner J, Reynolds J, Roman P. Organizational justice and job satisfaction: a test of three competing models. Soc Just Res. 2005;18(4):391–409. doi: 10.1007/s11211-005-8567-5

[99] Dilek H. A study on the effects of leadership styles and justice perception on organizational commitment, job satisfaction, and organizational citizenship behavior. Gebze (TR): Gebze Institute of Advanced Technology; 2005. https://tez.yok.gov.tr/UlusalTezMerkezi/

[100] Eker G. The effects of organizational justice dimensions on job satisfaction master’s thesis]. İzmir (TR): Dokuz Eylül University; 2006. https://tez.yok.gov.tr/UlusalTezMerkezi/

[101] Söyük S. The effect of organizational justice on job satisfaction: A study on nurses working in private hospitals in Istanbul. Istanbul (TR): Istanbul University; 2007. https://tez.yok.gov.tr/UlusalTezMerkezi/

[102] Yürür Ş. A study on the analysis of relationships between organizational justice, job satisfaction, and employees’ individual characteristics. Süleyman Demirel Univ J Fac Econ Adm Sci. 2008;13(2):295–312.

[103] Eroğlu ŞG. A study on organizational justice perception and job satisfaction. Denizli (TR): Pamukkale University. 2009. https://tez.yok.gov.tr/UlusalTezMerkezi/

[104] Colquitt JA, Conlon DE, Wesson MJ, Porter CO, Ng KY. Justice at the millennium: a meta-analytic review of 25 years of organizational justice research. J Appl Psychol. 2001;86(3):425–45. doi: 10.1037/0021-9010.86.3.425 11419803

[105] Greenberg J. Organizational justice: yesterday, today, and tomorrow. J Manag. 1990;16(2):399–432. doi: 10.1177/014920639001600208

[106] Karasar N. Scientific research methods. Ankara (TR): Nobel Publishing; 2011.

[107] Hoy WK, Tarter CJ. Organizational justice in schools: no justice without trust. Int J Educ Manag. 2004;18(4):250–9. doi: 10.1108/09513540410538831

[108] Hair JF, Black WC, Babin BJ, Anderson RE. Multivariate data analysis. 7th ed. Upper Saddle River (NJ): Pearson Education; 2010.

[109] Kline RB. Principles and practice of structural equation modeling. 2nd ed. New York (NY): Guilford Press; 2005.

[110] Tabachnick BG, Fidell LS. Using multivariate statistics. 6th ed. Boston (MA): Pearson; 2013.

[111] Baron RM, Kenny DA. The moderator-mediator variable distinction in social psychological research: conceptual, strategic, and statistical considerations. J Pers Soc Psychol. 1986;51(6):1173–82. doi: 10.1037//0022-3514.51.6.1173 3806354

[112] Brown TA. Confirmatory factor analysis for applied research. New York (NY): Guilford Press; 2006.

[113] Hu L, Bentler PM. Cutoff criteria for fit indexes in covariance structure analysis: conventional criteria versus new alternatives. Struct Equat Model: Multidiscip J. 1999;6(1):1–55. doi: 10.1080/10705519909540118

[114] Sun W, Liu X, Liu Y, Ding S, Jiang Y, Lv Z. The relationship between school organizational climate and teachers’ organizational citizenship behaviors: the mediating role of teaching efficacy and moderating role of optimistic traits. Behav Sci (Basel). 2024;14(12):1130. doi: 10.3390/bs14121130 39767271 PMC11673654

[115] Malque C, Smith J, Lee H. Perceived fairness and teacher commitment in private schools. Int J Educ Res. 2024;120:102–18.

[116] Tepper BJ, Taylor EC. Relationships among supervisors’ and subordinates’ procedural justice perceptions and organizational citizenship behaviors. Acad Manag J. 2003;46(1):97–105. doi: 10.2307/30040679

[117] Niehoff BP, Moorman RH. Justice as a mediator of the relationship between methods of monitoring and organizational citizenship behavior. Acad Manag J. 1993;36(3):527–56. doi: 10.2307/256591

